# Probiotic Implementation and Necrotizing Enterocolitis Risk in Preterm Infants

**DOI:** 10.1001/jamanetworkopen.2026.31601

**Published:** 2026-08-31

**Authors:** Ceren Imren, Vivian Jongejans, Wes Onland, Claudia M. G. Keyzer-Dekker, Jasper V. Been, Joep P. M. Derikx, Anton H. van Kaam, Sinno H. P. Simons, Jos W. R. Twisk, Chris H. P. van den Akker, Marijn J. Vermeulen

**Affiliations:** 1 Division of Neonatology, Department of Neonatal and Pediatric Intensive Care, Sophia Children’s Hospital, Erasmus Medical Center (MC), Rotterdam, the Netherlands; 2 Department of Pediatrics-Neonatology, Emma Children’s Hospital, Amsterdam University Medical Center (UMC), University of Amsterdam, Amsterdam, the Netherlands; 3 Amsterdam Reproduction and Development Research Institute, University of Amsterdam, Amsterdam, the Netherlands; 4 Department of Pediatric Surgery, Sophia Children’s Hospital, Erasmus MC, Rotterdam, the Netherlands; 5 Department of Obstetrics and Gynaecology, Sophia Children’s Hospital, Erasmus MC, Rotterdam, the Netherlands; 6 Department of Pediatric Surgery, Emma Children’s Hospital, Amsterdam UMC, University of Amsterdam, Amsterdam, the Netherlands; 7 Amsterdam Gastroenterology and Metabolism Research Institute, University of Amsterdam, Amsterdam, the Netherlands; 8 Department of Epidemiology and Data Science, Amsterdam UMC, Amsterdam, the Netherlands

## Abstract

**Importance:**

Despite their widespread use, uncertainty remains about the effectiveness and safety of probiotics for preventing necrotizing enterocolitis (NEC) in preterm infants. Recent regulatory warnings, including from the US Food and Drug Administration, have further emphasized the need for robust clinical data on both the benefits and risks of probiotics for this population.

**Objective:**

To evaluate the association between implementation of a multistrain probiotic formulation and NEC incidence in preterm infants in a high-risk neonatal intensive care unit (NICU) setting.

**Design, Setting, and Participants:**

This multicenter retrospective cohort study was conducted at 2 NICUs in the Netherlands. Preterm infants (<30 weeks of gestational age [GA], birth weight [BW] <1000 g, or both) born between January 1, 2018, and July 1, 2024, were compared before and after the implementation of routine probiotic supplementation. The probiotic protocol was introduced in October 2020 or March 2021, depending on the center.

**Exposure:**

Routine implementation of a multistrain probiotic formulation (*Bifidobacterium infantis* Bb-02, *Bifidobacterium lactis* BB-12, and *Streptococcus thermophilus* TH-4) as part of enteral feeding.

**Main Outcomes and Measures:**

The primary outcome was NEC incidence, classified as medical, surgical, or fatal. Secondary outcomes included all-cause mortality and mortality not associated with NEC. Primary and secondary outcomes were analyzed using generalized linear regression models, comparing preimplementation and postimplementation periods and adjusting for GA, sex, BW *z* score, 5-minute Apgar score, prenatal steroid use, and preterm premature rupture of membranes. Interrupted time-series analysis was performed as a sensitivity analysis to assess robustness against underlying temporal trends. Probiotic-related sepsis was monitored to assess safety.

**Results:**

This study included 1413 infants (742 males [52.5%]), stratified into a preimplementation group (n = 598) and a postimplementation group (n = 815). Baseline characteristics were comparable between groups (median GA, 27.7 weeks [IQR, 26.0-29.0 weeks] and 27.9 weeks [IQR, 26.4-29.0 weeks]; median BW, 950 g [IQR, 770-1170 g] and 975 g [IQR, 790-1190 g]). Following probiotic implementation, NEC incidence decreased from 11.9% (n = 71) to 5.3% (n = 43) (adjusted risk ratio [ARR], 0.49 [95% CI, 0.34-0.70]; *P* < .001). No statistically significant evidence of underlying time trends was detected. All-cause mortality remained stable for the preimplementation and postimplementation groups (70 [11.7%] vs 94 [11.5%]; ARR, 1.06 [95% CI, 0.82-1.39]; *P* = .64); non–NEC-associated mortality increased from 7.2% (n = 43) to 9.6% (n = 78) (ARR, 1.42 [95% CI, 1.01-1.98]; *P* = .04). One case of probiotic-associated sepsis was identified.

**Conclusions and Relevance:**

In this cohort study of preterm infants, implementation of a well-characterized multistrain probiotic was associated with a reduction in NEC incidence. This finding supports the use of the studied probiotic as an effective preventive strategy for NEC, although its effects on broader mortality outcomes remain uncertain.

## Key Points

**Question:**

Is routine administration of a well-characterized multistrain probiotic product associated with lower risk of necrotizing enterocolitis (NEC) among preterm infants?

**Findings:**

In this cohort study of 1413 preterm infants in 2 Dutch neonatal intensive care units, the implementation of routine probiotics was associated with a reduction in NEC incidence from 12% to 5%. One case of probiotic-associated sepsis was reported.

**Meaning:**

These findings suggest that routine use of multistrain probiotics may be an effective preventive strategy to reduce NEC in the high-risk neonatal care setting.

## Teaser

This cohort study compares necrotizing enterocolitis risk before and after implementation of routine probiotic supplementation among preterm infants in 2 Dutch neonatal intensive care units.

## Introduction

Necrotizing enterocolitis (NEC) is a severe gastrointestinal disease primarily affecting preterm infants and remains a major cause of neonatal morbidity and mortality.^1^ Its incidence is highest among extremely preterm infants.^2^ A key factor in NEC pathogenesis is dysbiosis—a microbial imbalance characterized by loss of beneficial bacteria (eg, bifidobacteria or lactobacilli), and overgrowth of potential pathogens like Enterobacteriaceae.^3^ This imbalance promotes intestinal inflammation and epithelial injury,^4^ increasing susceptibility to NEC.^5,6^ Probiotics may help prevent NEC by restoring microbial balance and suppressing pathogenic organisms.^4,7,8,9,10^ Individual strains may exert distinct functional properties, such as gut motility stimulation, mucosal barrier integrity enhancement, immunomodulation, and production of anti-inflammatory peptides, cytokines, and short-chain fatty acids.^11,12,13^ However, heterogeneity in probiotic products has contributed to inconsistent findings regarding their effectiveness.^9,10,14^ Strain specificity is therefore important when evaluating probiotic effectiveness and safety.

Safety concerns, including rare cases of probiotic-associated sepsis,^15,16^ have contributed to cautious global implementation in neonatal care. Although organizations such as the European Society for Paediatric Gastroenterology Hepatology and Nutrition (ESPGHAN)^9^ and the American Gastroenterological Association^17^ endorse specific strains, others, including the American Academy of Pediatrics (AAP), advise caution, particularly for infants weighing less than 1000 g.^16,18^ Since 2019, a combination of *Bifidobacterium infantis* Bb-02 (DSM 33361), *Bifidobacterium lactis* BB-12, and *Streptococcus thermophilus* TH-4 has been available in Europe, with documented effectiveness and safety in preterm infants.^8,9,19^ The current study evaluated NEC incidence following implementation of this probiotic regimen in 2 Dutch national intensive care units (NICUs).

## Methods

### Study Design

This multicenter, retrospective, observational cohort study was conducted at the level 4 NICUs of Erasmus Medical Center (MC) in Rotterdam and Amsterdam University Medical Center (UMC) in Amsterdam, the Netherlands. The study period spanned from January 1, 2018, to July 1, 2024, covering preimplementation and postimplementation phases of routine probiotic supplementation (Erasmus MC implementation: October 1, 2020; Amsterdam UMC implementation: March 1, 2021). This study was approved by the local ethics committees of Erasmus MC and Amsterdam UMC. Parental consent was waived due to the observational design. General consent for data use is routinely obtained at admission. Infants whose caregivers opted out were excluded. The study followed the Strengthening the Reporting of Observational Studies in Epidemiology (STROBE) reporting guideline.

Infants with a gestational age (GA) of less than 30 weeks, birth weight (BW) of less than 1000 g, or both were eligible for inclusion if they were born in, or transferred to, either NICU within 24 hours of birth. Exclusion criteria were gastrointestinal anomalies requiring surgery and death up to 48 hours after birth.

The primary analysis reflects an intention-to-treat approach based on period of birth relative to implementation. Therefore, it evaluates the effect of a NICU-level policy change rather than individual probiotic exposure.

### Probiotic Supplementation

The intervention involved implementation of a multistrain probiotic (ProPrems; Neobiomics) manufactured under good manufacturing practice standards and in accordance with European food law. Each batch was accompanied by certificates of strain identity, purity, viable counts at the end of shelf life, and antibiotic susceptibility and resistance profiles, in line with ESPGHAN recommendations.^8^

Each 0.5-g daily dose of the probiotic used contains a combined total of at least 10^9^ colony-forming units of *B infantis* Bb-02, *B lactis* BB-12, and *S thermophilus* TH-04. Once daily, trained personnel prepared the probiotic suspension by reconstituting the powder in 1.2 mL of sterile water under a laminar flow biosafety cabinet (class 2) or powder dust extraction cabinet. The suspension was transferred into enteral syringes and administered via gastric feeding tube within 2 hours of preparation, immediately before feeding.

Following implementation, all infants with a GA of less than 30 weeks, with BW of less than 1000 g, or both were prescribed the multistrain probiotic per national guidelines.^20^ Supplementation started once enteral feeds reached 2 mL per bolus (typically days 2-4 of life) and continued until 35 weeks postmenstrual age or NICU discharge, with temporary pauses during feeding cessation. The preimplementation cohort was not exposed to probiotics. All infants in both cohorts, in both hospitals, received either mother’s own milk or donor human milk, fortified with bovine-based fortifier once feeds reached 80 mL/kg/d. Nutritional protocols remained unchanged throughout the study periods.

### Outcome Measures

The primary outcome was the incidence of NEC. Secondary outcomes included all-cause mortality, non–NEC-associated mortality, focal intestinal perforation (FIP), late-onset sepsis (LOS), and time to full enteral feeding. Probiotic-associated sepsis was evaluated as a safety end point.

### Definitions

NEC was defined as modified Bell stage 2 or greater and additionally categorized as medical, surgical, or fatal NEC.^21^ Diagnoses were confirmed through review of clinical, surgical, radiology, and pathology reports by 2 researchers (C.v.d.A. and M.J.V), with consensus resolution of ambiguous cases. Medical NEC was defined as conservatively managed NEC (eg, antibiotics, cessation of enteral feeds), surgical NEC involved operative intervention, and fatal NEC included NEC-associated deaths, regardless of treatment. Each infant was classified according to the most severe NEC category; for example, infants with medical NEC who subsequently died from severe NEC-related sepsis were categorized as having fatal NEC.

LOS (>72 hours after birth) was defined as culture positive (gram positive or gram negative, intention to treat for ≥5 days) or culture negative (clinical sepsis symptoms, intention to treat for ≥5 days), using the National Institute of Child Health and Human Development Neonatal Research Network criteria.^22^ Isolated LOS occurred without gastrointestinal symptoms suggestive of NEC. Co-occurring NEC and LOS was classified as NEC.

FIP was defined as isolated intestinal perforation without generalized pneumatosis or necrosis.^23^ Time to full enteral feeding was defined as the interval from birth to the first day that parenteral amino acids and lipids were stopped, usually between 130 and 160 mL/kg/d of enteral feeding. This data point was missing for infants who were transferred or died before achieving this outcome.

### Reference Cohort

To account for potential time-related confounders coinciding with implementation (eg, changes in perinatal care practices), a parallel reference cohort was included. The reference cohort included infants born between 30 and 32 weeks of gestation with BW greater than 1000 g who did not receive probiotics per national guidelines. They were admitted to the same NICUs during the same period, ensuring comparability in clinical setting and care.

Although the more mature cohort had lower baseline risk for NEC and mortality,^24^ any underlying time-related changes in care may have affected both cohorts similarly. We hypothesized that no meaningful change in NEC incidence or mortality would be observed in the reference cohort following probiotic implementation. Reference infants were stratified into preimplementation and postimplementation groups based on their date of birth.

### Statistical Analysis

Potential confounders were identified based on clinical relevance and prior literature. These confounders included sex,^25^ multiple birth,^26^ GA,^27^ preterm premature rupture of membranes (PPROM),^28^ prenatal corticosteroid use (complete course: 2 dosages of betamethasone or 4 dosages of dexamethasone administered >24 hours before delivery),^29^ mode of delivery,^28^ 5-minute Apgar score,^30^ BW *z* score (based on the Fenton growth charts),^28,31^ season at birth,^32^ and early antibiotic exposure (<72 hours after birth).^33^ A directed acyclic graph was constructed to identify variables associated with both probiotic exposure and NEC (eFigure 1 in Supplement 1). Due to the limited number of outcome events, not all covariates could be included. Primary and secondary outcomes were analyzed using a generalized linear regression model with a log-link function and a binomial distribution, comparing postimplementation to preimplementation periods. Because exposure was defined by implementation period rather than individual probiotic receipt, these analyses estimate the effect of a routine implementation strategy under clinical conditions. Subgroup analyses were conducted similarly for medical, surgical, and fatal NEC. Results are presented as adjusted risk ratios (ARRs) and number needed to treat (NNT) with 95% CIs. Subgroup analyses for NEC incidence and mortality were also performed for extremely preterm infants.

Two complementary sensitivity analyses explored whether observed associations could be explained by background temporal trends. First, a segmented Poisson regression (interrupted time-series [ITS]) model was fitted, including time (continuous, expressed in 3-month intervals relative to the implementation point), probiotic implementation, and their interaction, with adjustment for GA, sex, and BW *z* score. This model estimated the preimplementation slope, immediate level change at implementation, and postimplementation slope. Second, parallel analyses in the reference cohort (nonexposed infants) used the same model to assess background trends unrelated to probiotic use.

Two-sided *P* < .05 was considered statistically significant. Analyses were conducted in RStudio, version 2023.12.14 (Posit Software), with R, version 4.3.2 (R Project for Statistical Computing).

## Results

### Cohort Characteristics

During the study period, 1413 infants (671 females [47.5%] and 742 males [52.5%]) born at a GA of less than 30 weeks or with a BW of less than 1000 g met the eligibility criteria (eFigure 2 in Supplement 1). They were stratified into a preimplementation group (n = 598) and a postimplementation group (n = 815). Baseline characteristics were generally similar between the preimplementation and postimplementation groups. The median GA was 27.7 weeks (IQR, 26.0-29.0 weeks) and 27.9 weeks (IQR, 26.4-29.0 weeks), and the median BW was 950 g (IQR, 770-1170 g) and 975 g (IQR, 790-1190 g), respectively. Distribution of sex, GA, and BW was comparable (Table 1). PPROM (99 [16.8%] vs 178 [22.8%]) and caesarean delivery (342 [57.2%] vs 517 [63.4%]) were more frequently observed after implementation compared with before.

**Table 1.** Perinatal Baseline Characteristics of the Study Groups^a^

Characteristic	Routine probiotic supplementation		SMD^b^
	Preimplementation group (n = 598)	Postimplementation group (n = 815)	
Sex			
Female	281/598 (47.0)	390/815 (47.9)	0.02
Male	317/598 (53.0)	425/815 (52.1)	
Gestational age, median (IQR), wk	27.7 (26.0-29.0)	27.9 (26.4-29.0)	0.06
Birth weight			
Median (IQR), g	950 (770-1170)	975 (790-1190)	0.05
*z* Score	0.1 (−0.7 to 0.7)	0.1 (−0.6 to 0.6)	0.002
Small for gestational age^c^	17/598 (2.8)	19/815 (2.3)	0.03
Extremely preterm^d^	318/598 (53.2)	427/815 (52.4)	0.02
Extremely low birth weight^e^	342/598 (57.2)	438/815 (53.7)	0.07
Multiple birth	125/598 (20.9)	154/815 (18.9)	0.05
PPROM	99/591 (16.8)	178/780 (22.8)	0.15
Prenatal steroid use, full course	355/582 (61.0)	439/743 (59.1)	0.04
Caesarean delivery	342/598 (57.2)	517/815 (63.4)	0.13
Apgar score, median (IQR), 5 min^f^	8 (7-9)	8 (7-9)	0.04
Early antibiotic administration	521/598 (87.1)	691/815 (84.8)	0.07

Abbreviations: PPROM, preterm premature rupture of membranes; SMD, standardized mean difference.

^a^ Data are shown as No. (%) of infants for categorical variables and as median (IQRs) for continuous variables for patients born before and after implementation of routine probiotic supplementation.

^b^ Standardized mean differences (SMDs) were calculated to assess group balance and are based on means and pooled SDs. An SMD of less than 0.1 was considered indicative of good balance.

^c^ Defined as birth weight for gestational age of less than −2 SD based on the Fenton growth charts.^31^

^d^ Defined as gestational age of less than 28 weeks.

^e^ Defined as birth weight of less than 1000 g.

^f^ Missing for 6 infants in the preimplementation group and 15 in the postimplementation group.

### Association Between Probiotic Implementation and NEC

Adherence to the probiotic protocol was 96.3% (n = 785 of 815). In the postimplementation group, 30 infants (3.7%) did not receive probiotics due to clinical contraindications, despite eligibility based on GA or BW criteria. NEC incidence decreased from 11.9% (n = 71) to 5.3% (n = 43) after implementation (Table 2), with lower proportions of medical, surgical, and fatal NEC observed. Decreases were seen for both Bell stages 3a and 3b.

**Table 2.** Unadjusted and Adjusted Associations of Probiotics With Outcome Measures^a^

Outcome measure	Routine probiotic supplementation		Risk ratio (95% CI)		*P* value^c^
	Preimplementation group (n = 598)	Postimplementation group (n = 815)	Crude	Adjusted^b^	
**Primary outcome**						
NEC Bell stage ≥2	71/598 (11.9)	43/815 (5.3)	0.44 (0.31-0.64)	0.49 (0.34-0.70)	<.001
Extremely premature infants	63/318 (19.8)	38/427 (8.9)	0.45 (0.31-0.65)	0.49 (0.34-0.72)	<.001
Medical NEC	23/598 (3.8)	15/815 (1.8)	0.48 (0.25-0.91)	0.50 (0.26-0.95)^d^	.03
Surgical NEC	21/598 (3.5)	12/815 (1.5)	0.42 (0.21-0.85)	0.44 (0.22-0.88)^d^	.02
Fatal NEC	27/598 (4.5)	16/815 (2.0)	0.43 (0.24-0.80)	0.47 (0.26-0.86)^d^	.01
NEC stage					
2A	15/598 (2.5)	11/815 (1.3)	0.54 (0.25-1.16)	0.56 (0.26-1.21)^d^	.14
2B	6/598 (1.0)	5/815 (0.6)	0.61 (0.19-1.99)	NA^e^	.41
3A	28/598 (4.7)	14/815 (1.7)	0.37 (0.19-0.69)	0.39 (0.21-0.73)^d^	.03
3B	22/598 (3.7)	13/815 (1.6)	0.43 (0.22-0.85)	0.46 (0.24-0.91)^d^	.02
**Secondary outcomes**						
All-cause mortality^f^	70/598 (11.7)	94/815 (11.5)	0.99 (0.74-1.32)	1.06 (0.82-1.39)	.64
Postnatal age at death, median (IQR)	10 (5-27)	11 (6-26)	NA	NA	.57
Extremely preterm infants	64/318 (20.1)	81/427 (19.0)	0.94 (0.70-1.26)	1.02 (0.77-1.34)	.91
Postnatal age at death for extremely preterm infants, median (IQR), d	10 (6-26)	11 (7-27)	NA	NA	.46
Non–NEC-associated mortality	43/598 (7.2)	78/815 (9.6)	1.33 (0.93-1.90)	1.42 (1.01-1.98)	.04
Postnatal age at death, median (IQR), d	7 (4-28)	9 (6-28)	NA	NA	.10
Extremely preterm infants	37/318 (11.6)	65/427 (15.2)	1.31 (0.90-1.91)	1.40 (0.97-2.01)	.07
Postnatal age at death for extremely preterm infants, median (IQR),d	7 (4-25)	9 (6-32)	NA	NA	.06
Late-onset sepsis	240/598 (40.1)	225/815 (27.6)	0.69 (0.59-0.80)	0.72 (0.63-0.83)	<.001
Gram negative	41/598 (6.9)	55/815 (6.7)	0.98 (0.67-1.45)	1.02 (0.69-1.50)	.92
Gram positive	94/598 (15.7)	111/815 (13.6)	0.87 (0.63-1.14)	0.92 (0.71-1.18)	.50
Culture negative	103/598 (17.2)	57/815 (7.0)	0.41 (0.30-0.55)	0.42 (0.31-0.57)	<.001
*Candida* species	2/598 (0.3)	1/815 (0.1)	NA^e^	NA^e^	.58
Probiotic sepsis	0	1/815 (0.1)^g^	NA^e^	NA^e^	>.99
Focal intestinal perforation	8/598 (1.3)	9/815 (1.1)	0.83 (0.32-2.13)	NA^e^	.70
Postnatal age at reaching full enteral feeding, median (IQR), d^h^	9 (8-11)	8 (7-10)	NA	NA	<.001

Abbreviations: NA, not applicable; NEC, necrotizing enterocolitis.

^a^ Unless indicated otherwise, data are presented as No. (%)/Total No. (%) of infants.

^b^ Adjusted for gestational age, sex, birth weight *z* score, Apgar score at 5 minutes, prenatal steroids, and premature prelabor rupture of membranes in a multivariable linear regression.

^c^ *P* value for adjusted analyses, or crude analyses, in which case risk ratios were calculated using the Fisher exact test.

^d^ Adjusted for gestational age.

^e^ Not applicable due to limited event counts.

^f^ Including fatal NEC.

^g^ Case of fatal NEC, infant died of severe pulmonary hemorrhage within hours after NEC and sepsis diagnosis.

^h^ Single-center data (preimplementation group: n = 244; postimplementation group: n = 356).

**Figure 1.**
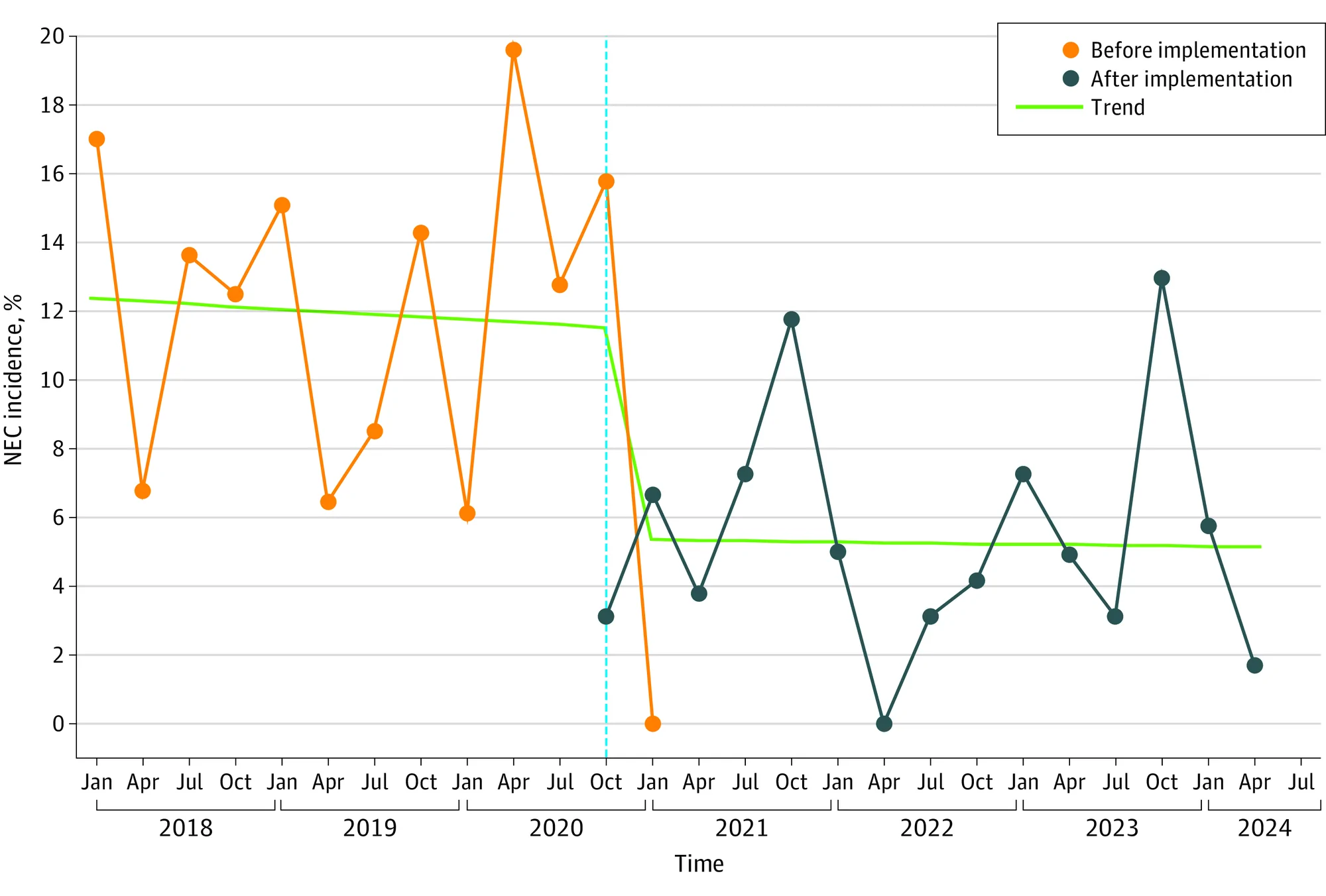
Line Graph of Necrotizing Enterocolitis (NEC) Rates Before and After Routine Probiotic Supplementation, 2018-2024 Implementation of routine probiotic supplementation began on October 1, 2020, at Erasmus Medical Center (MC) and on March 1, 2021, at Amsterdam University Medical Center (UMC). The overlapping lines around January 2021 reflect the transition period when Erasmus MC had already implemented supplementation, whereas Amsterdam UMC had not yet begun; thus, this period also contains a lower number of infants at risk. The green line represents the fitted trend line for the segmented Poisson interrupted time-series regression model.

**Figure 2.**
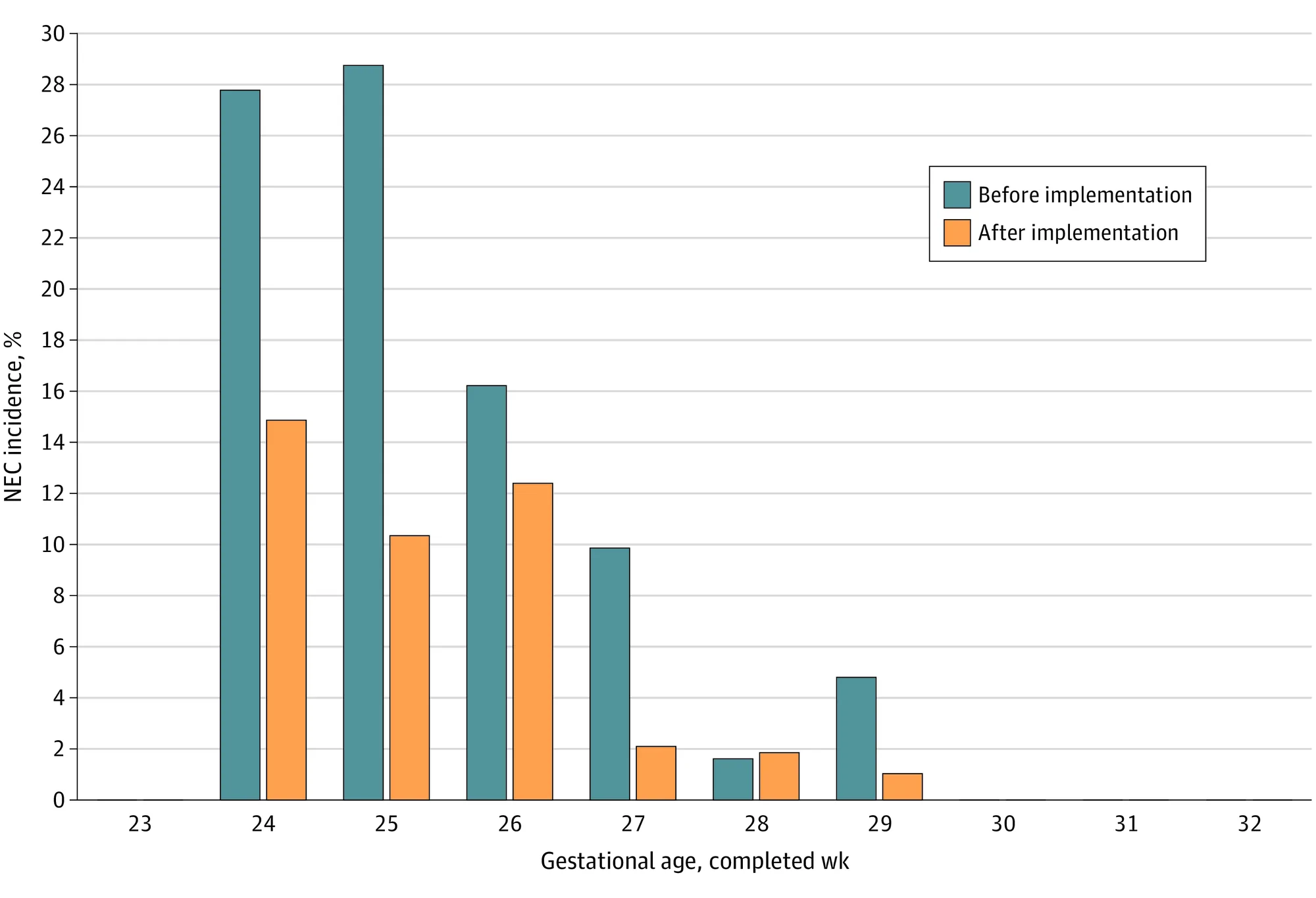
Bar Graph of Necrotizing Enterocolitis (NEC) Rates by Gestational Age NEC rates are shown as the proportions of the total number of infants in each group per completed week of gestation. Infants are grouped by whole weeks (eg, *x* = 24 includes all infants with a gestational age of 24 weeks plus 0-6 days).

Probiotic implementation remained associated with a 51% lower risk of NEC after adjusting for GA, sex, BW *z* score, 5-minute Apgar score, prenatal steroids, and PPROM (ARR, 0.49 [95% CI, 0.34-0.70]; *P* < .001) (Table 2). The NNT corresponding to this association was 15 (95% CI, 10-28). Similar associations were observed for medical NEC (ARR, 0.50 [95% CI, 0.26-0.95]; *P* = .03), surgical NEC (ARR, 0.44 [95% CI, 0.22-0.88]; *P* = .02), and fatal NEC (ARR, 0.47 [95% CI, 0.26-0.86]; *P* = .01), as well as among extremely preterm infants (ARR, 0.49 [95% CI, 0.34-0.72]; *P* < .001). Figure 1 shows the incidence over time for both the preimplementation and postimplementation periods. Lower NEC rates after implementation were observed across all GA weeks (Figure 2).

The preimplementation time trend in the ITS analysis did not indicate a temporal change in NEC incidence prior to probiotic implementation (β = 0.01; risk ratio [RR], 1.01 [95% CI, 0.94-1.08]; *P* = .80) (eTable 1 in Supplement 1). The finding for probiotic implementation suggested a decline in NEC incidence at the level of the intervention period (β = −0.76; RR, 0.47 [95% CI, 0.21-1.00]; *P* = .05) (Figure 1). After implementation, we did not observe evidence of a change in slope (β = −0.01; RR, 0.99 [95% CI, 0.89-1.09]; *P* = .79).

### Reference Cohort

Baseline characteristics of the reference cohort (n = 771 infants; 343 females [44.5%] and 428 males [55.5%]) were comparable between the preimplementation (n = 319) and postimplementation (n = 425) periods (eTable 2 in Supplement 1). The median GA was 31.0 weeks (IQR, 30.4-31.6 weeks) and 31.0 weeks (IQR, 30.6-31.4 weeks), and the median BW was 1540 g (IQR, 1320-1710 g) and 1560 g (IQR, 1350-1725 g) for the preimplementation and postimplementation groups, respectively. NEC incidence was lower after implementation, although not statistically significant (6 [5.0%] vs 12 [2.7%]; *P* = .12) (eTable 3 and eFigure 3 in Supplement 1). ITS analysis did not detect evidence of a preimplementation trend (β = 0.01; RR, 1.01 [95% CI, 0.87-1.18]; *P* = .88) (eTable 1 in Supplement 1), an immediate change at implementation (β = −0.78; RR, 0.46 [95% CI, 0.09-2.20]; *P* = .33), or a change in postimplementation slope (β = 0.02; RR, 1.02 [95% CI, 0.82-1.25]; *P* = .93). All-cause mortality (9 [2.8%] vs 11 [2.4%]), non–NEC-associated mortality (6 [1.9%] vs 9 [2.0%]), and LOS (36 [11.3%] vs 37 [8.2%]) remained unchanged.

### Mortality

All-cause mortality was similar before and after probiotic implementation (70 [11.7%] vs 94 [11.5%]; ARR, 1.06 [95% CI, 0.82-1.39]; *P* = .64) (Table 2), with comparable median postnatal age at death (10 days [IQR, 5-27 days] vs 11 days [IQR, 6-26 days]). Mortality among extremely preterm infants was similarly unchanged (ARR, 1.02 [95% CI, 0.77-1.34]; *P* = .91).

Non–NEC-associated mortality was higher in the postimplementation period (43 of 598 [7.2%] vs 78 of 815 [9.6%]) and was significant after adjustment (ARR, 1.42 [95% CI, 1.01-1.98]; *P* = .04). The median postnatal age at death increased from 7 days (IQR, 4-28 days) to 9 days (IQR, 6-28 days) (*P* = .10). A similar pattern was observed among extremely preterm infants (37 of 318 [11.6%] vs 65 of 427 [15.2%]; ARR, 1.40 [95% CI, 0.97-2.01]; *P* = .07), with postnatal age at death increasing from 7 days (IQR, 4-25 days) to 9 days (IQR, 6-32 days) (*P* = .06). Higher non–NEC-associated mortality was reflected across multiple causes that appeared largely unrelated to probiotic use, including death due to persistent pulmonary hypertension of the newborn (PPHN), perinatal asphyxia, and cardiac or neurologic causes (Table 3). Four additional deaths due to FIP occurred after implementation; however, the overall FIP incidence was unchanged.

**Table 3.** Causes of Non–NEC-Associated Death^a^

Cause	No. (%) of non–NEC-associated deaths	
	Preimplementation group (n = 598)	Postimplementation group (n = 815)
Sepsis	16 (2.7)	24 (2.9)
Respiratory	11 (1.8)	12 (1.5)
PPHN	5 (0.8)	10 (1.2)
Cardiac	0	3 (0.4)
Neurologic	7 (1.2)	13 (1.6)
Abdominal other than NEC	0	5 (0.6)^a^
Metabolic, renal, hepatic	3 (0.5)	5 (0.6)
Perinatal asphyxia	0	5 (0.6)
Unknown etiology	1 (0.2)	1 (0.1)
Total No. of deaths	43 (7.2)	78 (9.6)

Abbreviations: NEC, necrotizing enterocolitis; PPHN, persistent pulmonary hypertension of the newborn.

^a^ Focal intestinal perforation (n = 4) and gastrointestinal obstruction not further specified (n = 1).

### Other Secondary Outcomes

LOS was lower in the postimplementation period (240 [40.1%] vs 225 [27.6%]; ARR, 0.72 [95% CI, 0.63-0.83]; *P* < .001) (Table 2), mainly due to fewer culture-negative episodes (103 [17.2%] vs 57 [7.0%]; *P* < .001). FIP incidence was similar between periods (8 [1.3%] vs 9 [1.1%]). The median time to full enteral feeding was shorter after implementation (9 days [IQR, 8-11 days] vs 8 days [IQR, 7-10 days]; *P* < .001). One case (0.1%) of probiotic sepsis occurred. The infant suddenly deteriorated, and radiography showed extensive pneumatosis. The blood culture grew *B infantis*. Within hours, the infant died due to severe pulmonary hemorrhage despite intensive support.

## Discussion

In this observational study conducted in the neonatal care setting, implementation of probiotics was associated with a 51% lower NEC incidence, which is consistent across medical, surgical, and fatal NEC and similar among extremely preterm infants compared with more mature preterm infants. ITS analysis did not provide evidence that underlying temporal changes explained the observed reduction in NEC incidence. Probiotic implementation was not associated with lower all-cause mortality due to a higher non–NEC-related mortality rate after implementation, which did not appear directly attributable to probiotic use. One case of probiotic-associated sepsis occurred and coincided with fulminant NEC.

Our findings align with those from a previous trial using the same probiotic formulation, which reported a comparable lower NEC risk (RR, 0.46 [95% CI, 0.23-0.93]) among infants with a GA of less than 32 weeks and weighing less than 1500 g.^34^ The comparable magnitude of association supports the formulation’s effectiveness in clinical practice. Our results also align with a 2023 meta-analysis reporting lower NEC risk with probiotics overall (RR, 0.54 [95% CI, 0.46-0.65]), despite heterogeneity in strains, protocols, and populations.^10^ Importantly, our cohort included a substantial number of extremely preterm infants (<28 weeks of GA), enabling subgroup analysis that showed similar effect sizes. Existing evidence among extremely preterm infants remains mixed^27^: the 2023 Cochrane review found no association (RR, 0.92 [95% CI, 0.69-1.22]),^10^ whereas a 2025 meta-analysis, including nonrandomized studies, reported lower risk (RR. 0.80 [95% CI, 0.68-0.93]).^35^ This variability may reflect heterogeneity in probiotic strains, combinations, NICU practices, and analytic methods.

ITS analysis did not detect evidence that underlying temporal trends accounted for the observed reduction in NEC incidence, although we cannot exclude modest secular changes. The results from the ITS analyses in both the study and reference cohorts strengthen the interpretation that probiotic implementation contributed to the observed reduction. Importantly, potential cross-colonization into the reference cohort may have resulted in indirect probiotic exposure, which could have attenuated differences between groups.^36^ Enteral nutrition protocols remained unchanged during the study period, reducing the likelihood that concurrent feeding-related interventions explained the observed findings. Nonetheless, residual confounding cannot be excluded, given the limitations of disentangling intervention effects from time-related changes in nonrandomized designs.

Despite the lower NEC incidence, all-cause mortality did not change in this study. Prior studies have reported mixed findings, with mortality reductions observed in very preterm infants, but not consistently among extremely preterm infants.^10^ Discrepancies may reflect differences in probiotic formulations, baseline mortality risks, and statistical power.

In this study, non–NEC**-**associated mortality increased from 7.2% before implementation to 9.6% after implementation, with deaths distributed across multiple causes, including PPHN, perinatal asphyxia, cardiac, and neurologic causes, which are unlikely to be directly attributable to probiotic administration. Additionally, deaths due to FIP were higher in the postimplementation group (n = 0 vs 4), although the overall FIP incidence remained unchanged. It remains speculative whether the increase in non–NEC-associated mortality reflects unmeasured differences in case mix, illness severity, or changes in care practices including or excluding probiotic implementation. However, this finding underscores the need for ongoing safety surveillance and further research.

LOS rates decreased significantly in this study, primarily due to fewer culture-negative episodes. This finding may reflect improved gut barrier function and reduced feeding intolerance and systemic inflammation, leading to fewer episodes of clinical instability without proven infection. Prior meta-analyses have reported similar overall reductions in LOS with probiotics,^37,38^ although none specifically addressed culture-negative cases. Our definition of culture-negative sepsis reflects clinical decision-making rather than a uniform pathophysiologic entity, highlighting the need for prospective studies with standardized sepsis definitions and detailed physiologic data.

The routine use of single aerobic pediatric blood culture bottles, which is standard practice in many NICUs, may reduce detection of low bacterial loads of anaerobic organisms such as bifidobacteria. Underdetection of probiotic-associated bacteremia cannot be fully excluded, although rates of culture-negative sepsis point in the opposite likelihood. Additionally, the probiotic product used in our study also contains *S thermophilus*, which grows well under aerobic conditions but was never detected in blood cultures, providing indirect evidence against common occult probiotic bacteremia with undetected anaerobes.

In fact, one adverse event potentially associated with probiotics occurred in 1 patient in this study: a case of fulminant NEC in which the aerobic blood culture grew *B infantis*. Although causality remains uncertain, this event underscores the importance of careful monitoring. This single case among more than 800 exposed infants suggests such events are rare, consistent with prior reported incidences of probiotic sepsis among *Bifidobacterium*-containing probiotics.^39^ Although recent US Food and Drug Administration (FDA) and AAP statements urge caution due to regulatory and safety concerns,^15,18^ these are debated as well.^16,40^ Our results and growing international evidence support the safe and effective use of selected, well-characterized probiotic strains.

### Strengths and Limitations

The strengths of this study include its large, multicenter cohort, use of a parallel reference cohort, and adjustment for relevant covariates, with explicit adjustment for temporal trends, reducing time-related confounding. Uniform use of human milk rather than formula minimized nutritional confounding.

Study limitations include the retrospective design, residual susceptibility to secular confounding inherent to pre-to-post comparisons, and potential unmeasured confounding. In addition, exposure was defined according to implementation period rather than confirmed individual probiotic receipt, such that analyses estimate the association of a probiotic implementation strategy under clinical conditions rather than individual-level efficacy. However, adherence to the probiotic protocol was high (96.3%) following implementation. The relatively high NEC incidence before implementation, despite breast feeding protocols already in place,^41,42,43^ may reflect differences in case mix or diagnostic practices underscoring the limitations of retrospective comparisons. Future research should focus on harmonizing probiotic formulations across studies, understanding strain-specific effects, and identifying subgroups most likely to benefit. Randomized trials are warranted to more precisely estimate the effectiveness and safety of specific probiotic strains in extremely preterm infants.

## Conclusions

In this cohort study, routine supplementation with a well-characterized probiotic formulation (*B infantis* Bb-02, *B lactis* BB-12, and *S thermophilus* TH-04) was associated with lower NEC incidence among very and extremely preterm infants. These findings provide clinical evidence supporting the preventive benefit of this specific formulation. However, the observed increase in non–NEC-associated mortality warrants cautious interpretation and further investigation. Future research should aim to harmonize probiotic formulations, clarify strain-specific effects, and identify subpopulations most likely to benefit.

## Notes

Supplement 1. **eFigure 1.** Directed Acyclic Graph **eFigure 2.** Flowchart of Inclusion **eFigure 3.** Necrotizing Enterocolitis (NEC) Rates Over Time, by 3-Month Intervals for the Reference Cohort **eTable 1.** Interrupted Time-Series Results for the Study and Reference Cohorts **eTable 2.** Perinatal Baseline Characteristics for the Reference Cohort **eTable 3.** Unadjusted Outcomes for the Reference Cohort

Supplement 2.
Data Sharing Statement

## References

[1] Imren C, Vlug LE, de Koning BAE, . Necrotizing enterocolitis in a Dutch cohort of very preterm infants: prevalence, mortality, and long-term outcomes. Eur J Pediatr Surg. 2022;32(1):111-119. doi:10.1055/s-0041-1741544 35008115

[2] Battersby C, Santhalingam T, Costeloe K, Modi N. Incidence of neonatal necrotising enterocolitis in high-income countries: a systematic review. Arch Dis Child Fetal Neonatal Ed. 2018;103(2):F182-F189. doi:10.1136/archdischild-2017-313880 29317459

[3] Rodríguez JM, Murphy K, Stanton C, . The composition of the gut microbiota throughout life, with an emphasis on early life. Microb Ecol Health Dis. 2015;26:26050. doi:10.3402/mehd.v26.26050 25651996 PMC4315782

[4] Duess JW, Sampah ME, Lopez CM, . Necrotizing enterocolitis, gut microbes, and sepsis. Gut Microbes. 2023;15(1):2221470. doi:10.1080/19490976.2023.2221470 37312412 PMC10269420

[5] Morowitz MJ, Poroyko V, Caplan M, Alverdy J, Liu DC. Redefining the role of intestinal microbes in the pathogenesis of necrotizing enterocolitis. Pediatrics. 2010;125(4):777-785. doi:10.1542/peds.2009-3149 20308210

[6] Stewart CJ, Marrs EC, Magorrian S, . The preterm gut microbiota: changes associated with necrotizing enterocolitis and infection. Acta Paediatr. 2012;101(11):1121-1127. doi:10.1111/j.1651-2227.2012.02801.x 22845166

[7] Gomaa EZ. Human gut microbiota/microbiome in health and diseases: a review. Antonie Van Leeuwenhoek. 2020;113(12):2019-2040. doi:10.1007/s10482-020-01474-7 33136284

[8] van den Akker CHP, van Goudoever JB, Shamir R, . Probiotics and preterm infants: a position paper by the European Society for Paediatric Gastroenterology Hepatology and Nutrition Committee on Nutrition and the European Society for Paediatric Gastroenterology Hepatology and Nutrition Working Group for Probiotics and Prebiotics. J Pediatr Gastroenterol Nutr. 2020;70(5):664-680. doi:10.1097/MPG.0000000000002655 32332478

[9] van den Akker CHP, van Goudoever JB, Szajewska H, ; ESPGHAN Working Group for Probiotics, Prebiotics & Committee on Nutrition. Probiotics for preterm infants: a strain-specific systematic review and network meta-analysis. J Pediatr Gastroenterol Nutr. 2018;67(1):103-122. doi:10.1097/MPG.0000000000001897 29384838

[10] Sharif S, Meader N, Oddie SJ, Rojas-Reyes MX, McGuire W. Probiotics to prevent necrotising enterocolitis in very preterm or very low birth weight infants. Cochrane Database Syst Rev. 2023;7(7):CD005496. 37493095 10.1002/14651858.CD005496.pub6 PMC10370900

[11] Rose EC, Odle J, Blikslager AT, Ziegler AL. Probiotics, prebiotics and epithelial tight junctions: a promising approach to modulate intestinal barrier function. Int J Mol Sci. 2021;22(13):6729. doi:10.3390/ijms22136729 34201613 PMC8268081

[12] Sadeghpour Heravi F, Hu H. Bifidobacterium: host-microbiome interaction and mechanism of action in preventing common gut-microbiota-associated complications in preterm infants: a narrative review. Nutrients. 2023;15(3):709. doi:10.3390/nu15030709 36771414 PMC9919561

[13] Samara J, Moossavi S, Alshaikh B, . Supplementation with a probiotic mixture accelerates gut microbiome maturation and reduces intestinal inflammation in extremely preterm infants. Cell Host Microbe. 2022;30(5):696-711.e5. doi:10.1016/j.chom.2022.04.005 35550672

[14] Wang Y, Florez ID, Morgan RL, . Probiotics, prebiotics, lactoferrin, and combination products for prevention of mortality and morbidity in preterm infants: a systematic review and network meta-analysis. JAMA Pediatr. 2023;177(11):1158-1167. doi:10.1001/jamapediatrics.2023.3849 37782505 PMC10546299

[15] Warning regarding use of probiotics in preterm infants. US Food and Drug Administration. September 29, 2023. Accessed July 1, 2025. https://www.fda.gov/media/172606/download

[16] van den Akker CHP, Embleton ND, Lapillonne A, . Reevaluating the FDA’s warning against the use of probiotics in preterm neonates: a societal statement by ESPGHAN and EFCNI. J Pediatr Gastroenterol Nutr. 2024;78(6):1403-1408. doi:10.1002/jpn3.12204 38572770

[17] Su GL, Ko CW, Bercik P, . AGA clinical practice guidelines on the role of probiotics in the management of gastrointestinal disorders. Gastroenterology. 2020;159(2):697-705. doi:10.1053/j.gastro.2020.05.059 32531291

[18] Poindexter B; COMMITTEE ON FETUS AND NEWBORN. Use of probiotics in preterm infants. Pediatrics. 2021;147(6):B. doi:10.1542/peds.2021-051485 34031231

[19] Dai Y, Yu Q, Zhang F, . Effect of probiotics on necrotizing enterocolitis in preterm infants: a network meta-analysis of randomized controlled trials. BMC Pediatr. 2025;25(1):237. doi:10.1186/s12887-025-05469-z 40140789 PMC11948853

[20] N3 Aanbeveling 2021 Probiotica bij prematuur geboren neonaten opgenomen op de NICU (Dutch National Recommendation on Probiotics in Preterm Infants). Neonatology Network. Accessed July 1, 2025. https://neonatology.eu/sites/default/files/u180/n3_aanbeveling_2021_probiotica_0.pdf

[21] Walsh MC, Kliegman RM. Necrotizing enterocolitis: treatment based on staging criteria. Pediatr Clin North Am. 1986;33(1):179-201. doi:10.1016/S0031-3955(16)34975-6 3081865 PMC7131118

[22] Boghossian NS, Page GP, Bell EF, ; Eunice Kennedy Shriver National Institute of Child Health and Human Development Neonatal Research Network. Late-onset sepsis in very low birth weight infants from singleton and multiple-gestation births. J Pediatr. 2013;162(6):1120-1124, 1124.e1. doi:10.1016/j.jpeds.2012.11.089 23324523 PMC3633723

[23] Krishnan P, Lotfollahzadeh S. Spontaneous intestinal perforation of the newborn. Updated June 3, 2023. Accessed August 11, 2026. https://www.ncbi.nlm.nih.gov/books/NBK585031/36251805

[24] Walsh MC, Bell EF, Kandefer S, . Neonatal outcomes of moderately preterm infants compared to extremely preterm infants. Pediatr Res. 2017;82(2):297-304. doi:10.1038/pr.2017.46 28419085 PMC5552426

[25] Pijpers AGH, Imren C, van Varsseveld OC, . Risk factors for 30-day mortality in patients with surgically treated necrotizing enterocolitis: a multicenter retrospective cohort study. Eur J Pediatr Surg. 2025;35(4):332-340. doi:10.1055/a-2536-4757 40118095 PMC12245516

[26] Vedel C, Larsen H, Holmskov A, . Neonatal complications and neurophysiological development in twins—a long-term follow-up study. J Matern Fetal Neonatal Med. 2022;35(2):372-378. doi:10.1080/14767058.2020.1718647 31986942

[27] Kruth SS, Willers C, Persad E, Sjöström ES, Lagerström SR, Rakow A. Probiotic supplementation and risk of necrotizing enterocolitis and mortality among extremely preterm infants—the Probiotics in Extreme Prematurity in Scandinavia (PEPS) trial: study protocol for a multicenter, double-blinded, placebo-controlled, and registry-based randomized controlled trial. Trials. 2024;25(1):259. doi:10.1186/s13063-024-08088-8 38610034 PMC11015611

[28] Samuels N, van de Graaf RA, de Jonge RCJ, Reiss IKM, Vermeulen MJ. Risk factors for necrotizing enterocolitis in neonates: a systematic review of prognostic studies. BMC Pediatr. 2017;17(1):105. doi:10.1186/s12887-017-0847-3 28410573 PMC5391569

[29] Roberts D, Dalziel S. Antenatal corticosteroids for accelerating fetal lung maturation for women at risk of preterm birth. Cochrane Database Syst Rev. 2006;(3):CD004454. doi:10.1002/14651858.CD004454.pub2 16856047

[30] Seeman SM, Mehal JM, Haberling DL, Holman RC, Stoll BJ. Infant and maternal risk factors related to necrotising enterocolitis-associated infant death in the United States. Acta Paediatr. 2016;105(6):e240-e246. doi:10.1111/apa.13390 26946352

[31] Fenton TR, Kim JH. A systematic review and meta-analysis to revise the Fenton growth chart for preterm infants. BMC Pediatr. 2013;13:59. doi:10.1186/1471-2431-13-59 23601190 PMC3637477

[32] Samuels N, van de Graaf R, Been JV, . Necrotising enterocolitis and mortality in preterm infants after introduction of probiotics: a quasi-experimental study. Sci Rep. 2016;6:31643. doi:10.1038/srep31643 27545195 PMC4992873

[33] Neu J. Prevention of necrotizing enterocolitis. Clin Perinatol. 2022;49(1):195-206. doi:10.1016/j.clp.2021.11.012 35210001

[34] Jacobs SE, Tobin JM, Opie GF, ; ProPrems Study Group. Probiotic effects on late-onset sepsis in very preterm infants: a randomized controlled trial. Pediatrics. 2013;132(6):1055-1062. doi:10.1542/peds.2013-1339 24249817

[35] Söderquist Kruth S, Persad E, Rakow A. Probiotic supplements effect on feeding tolerance, growth and neonatal morbidity in extremely preterm infants: a systematic review and meta-analysis. Nutrients. 2025;17(7):1228. doi:10.3390/nu17071228 40218986 PMC11990243

[36] Costeloe K, Hardy P, Juszczak E, Wilks M, Millar MR; Probiotics in Preterm Infants Study Collaborative Group. Bifidobacterium breve BBG-001 in very preterm infants: a randomised controlled phase 3 trial. Lancet. 2016;387(10019):649-660. doi:10.1016/S0140-6736(15)01027-2 26628328

[37] Aceti A, Maggio L, Beghetti I, ; Italian Society of Neonatology. Probiotics prevent late-onset sepsis in human milk-fed, very low birth weight preterm infants: systematic review and meta-analysis. Nutrients. 2017;9(8):904. doi:10.3390/nu9080904 28829405 PMC5579697

[38] Rao SC, Athalye-Jape GK, Deshpande GC, Simmer KN, Patole SK. Probiotic supplementation and late-onset sepsis in preterm infants: a meta-analysis. Pediatrics. 2016;137(3):e20153684. doi:10.1542/peds.2015-3684 26908700

[39] Alshaikh BN, Ting J, Lee S, ; Canadian Neonatal Network Investigators. Effectiveness and risks of probiotics in preterm infants. Pediatrics. 2025;155(3):e2024069102. doi:10.1542/peds.2024-069102 39933567

[40] Embleton ND, Berrington J, Clarke P, . Probiotics for preterm infants and the recent FDA alert in the USA. Arch Dis Child Fetal Neonatal Ed. 2024;109(6):e1. doi:10.1136/archdischild-2023-326580 38123949

[41] Heida FH, Stolwijk L, Loos MH, . Increased incidence of necrotizing enterocolitis in the Netherlands after implementation of the new Dutch guideline for active treatment in extremely preterm infants: results from three academic referral centers. J Pediatr Surg. 2017;52(2):273-276. doi:10.1016/j.jpedsurg.2016.11.024 27923478

[42] Han SM, Hong CR, Knell J, . Trends in incidence and outcomes of necrotizing enterocolitis over the last 12 years: a multicenter cohort analysis. J Pediatr Surg. 2020;55(6):998-1001. doi:10.1016/j.jpedsurg.2020.02.046 32173122

[43] Stoll BJ, Hansen NI, Bell EF, ; Eunice Kennedy Shriver National Institute of Child Health and Human Development Neonatal Research Network. Trends in care practices, morbidity, and mortality of extremely preterm neonates, 1993-2012. JAMA. 2015;314(10):1039-1051. doi:10.1001/jama.2015.10244 26348753 PMC4787615

