# Supplementary material for: Probiotic Implementation and Necrotizing Enterocolitis Risk in Preterm Infants
Source: JAMA Netw Open. 2026 Aug 31;9(8):e2631601. doi: 10.1001/jamanetworkopen.2026.31601 (PMC13531319; doi:10.1001/jamanetworkopen.2026.31601)
Supplement: Supplementary file 1 [file jamanetwopen-e2631601-s001.pdf]

Supplementary Online Content

Imren C, Jongejans V, Onland W, et al. Probiotic implementation and necrotizing enterocolitis risk in preterm infants. *JAMA Netw Open*. 2026;9(8):e2631601. doi:10.1001/jamanetworkopen.2026.31601

eFigure 1. Directed Acyclic Graph

eFigure 2. Flowchart of Inclusion

eFigure 3. Necrotizing Enterocolitis (NEC) Rates Over Time, by 3-Month Intervals for the Reference Cohort

eTable 1. Interrupted Time-Series Results for the Study and Reference Cohorts

eTable 2. Perinatal Baseline Characteristics for the Reference Cohort

eTable 3. Unadjusted Outcomes for the Reference Cohort

This supplementary material has been provided by the authors to give readers additional information about their work.

© 2026 Imren C et al. *JAMA Network Open*.

eFigure 1. Directed Acyclic Graph

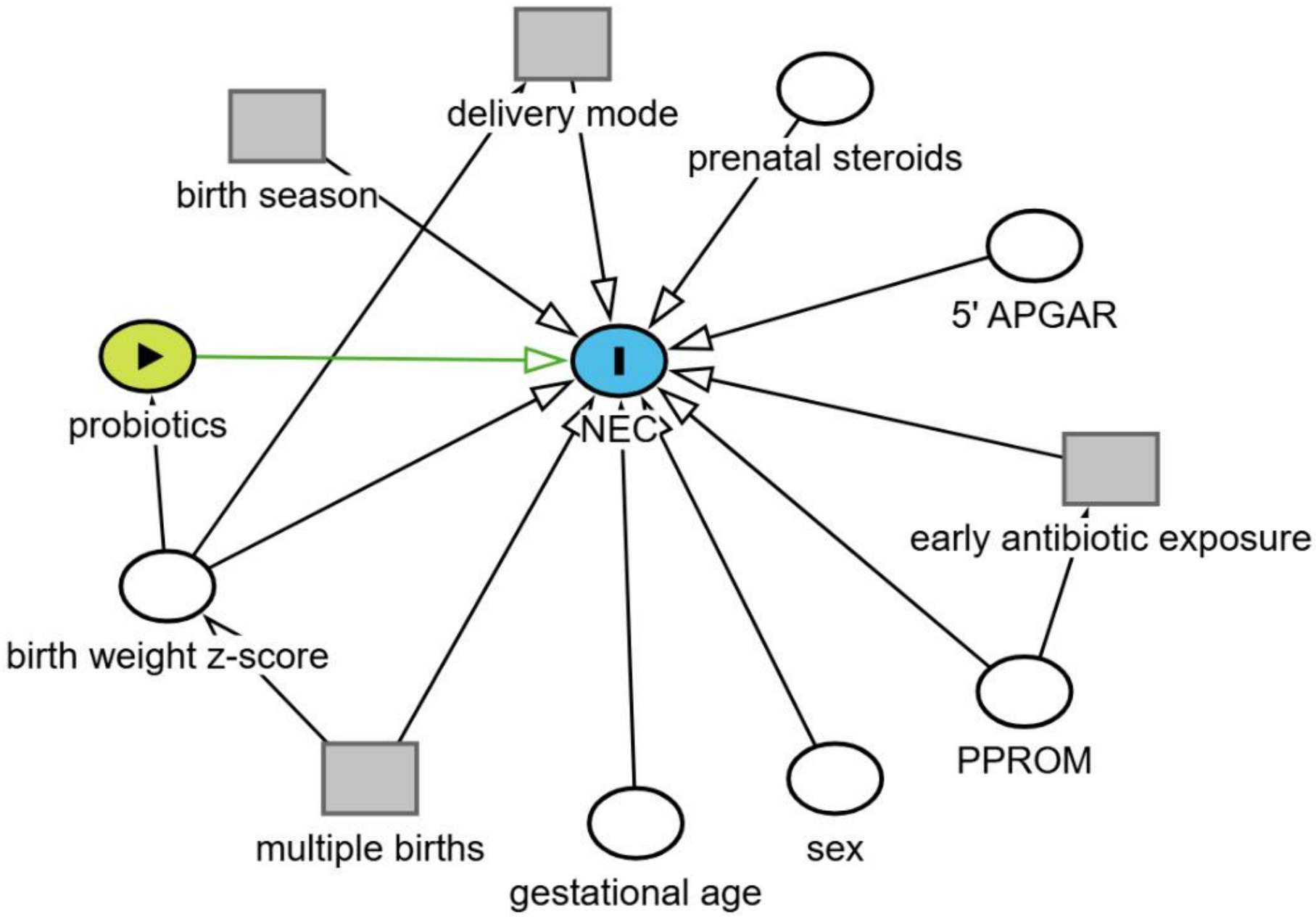

Directed acyclic graph (DAG) visualizing covariates selected based on their potential relationship with both probiotic exposure and NEC. Potential covariates were retained in the final model if their removal changed the regression coefficient by >10%, indicating confounding. Legend: Green circle exposure; blue circle outcome; white circle adjusted covariate; grey square unadjusted variable. Figure was created using DAGitty online software; www.daggity.net.

© 2026 Imren C et al. *JAMA Network Open*.

eFigure 2. Flowchart of Inclusion

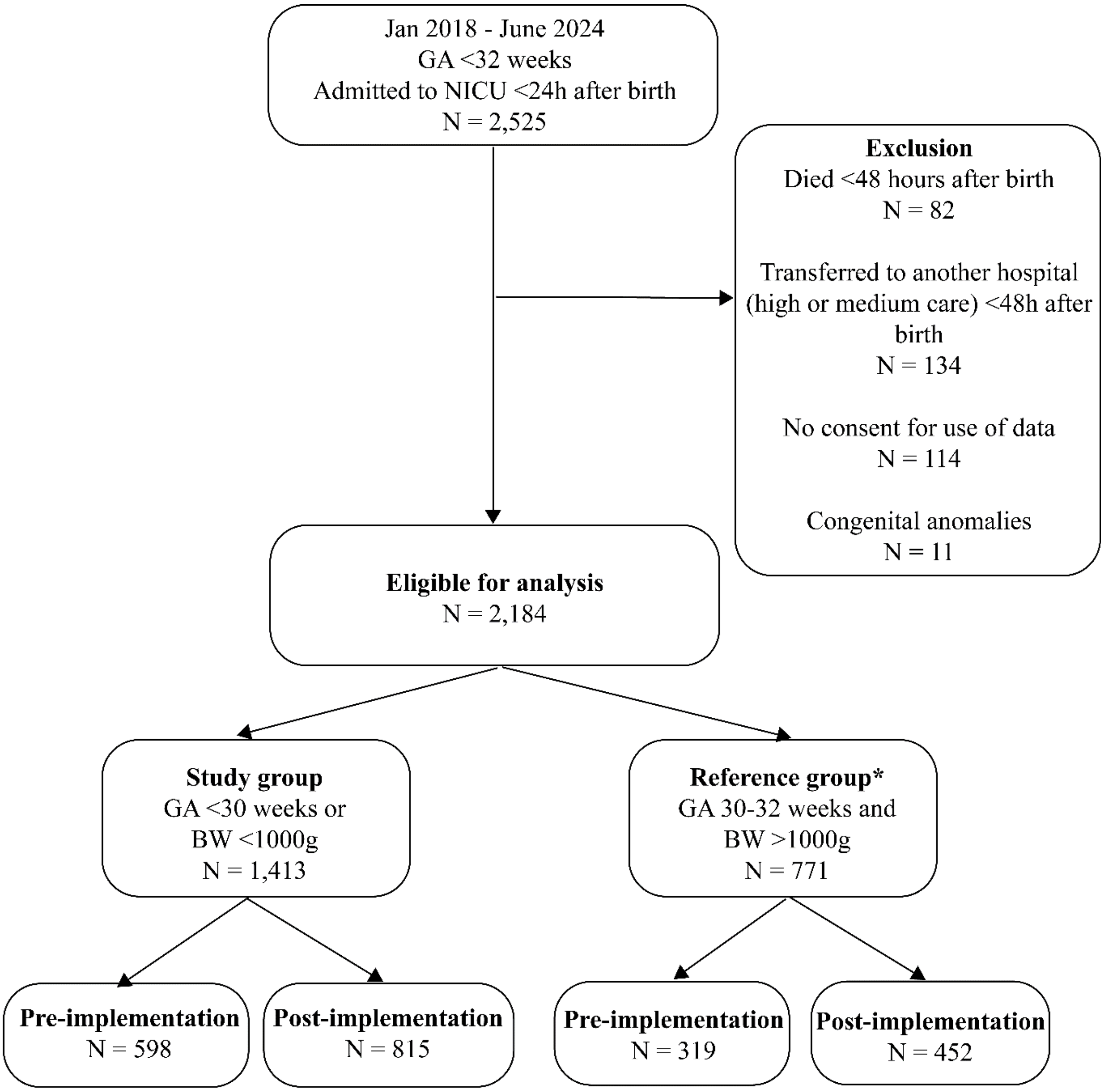

*No indication for probiotics.
Abbreviations: GA: gestational age; BW: birth weight; NICU: neonatal intensive care unit.

© 2026 Imren C et al. *JAMA Network Open*.

eFigure 3. Necrotizing Enterocolitis (NEC) Rates Over Time, by 3-Month Intervals for the Reference Cohort

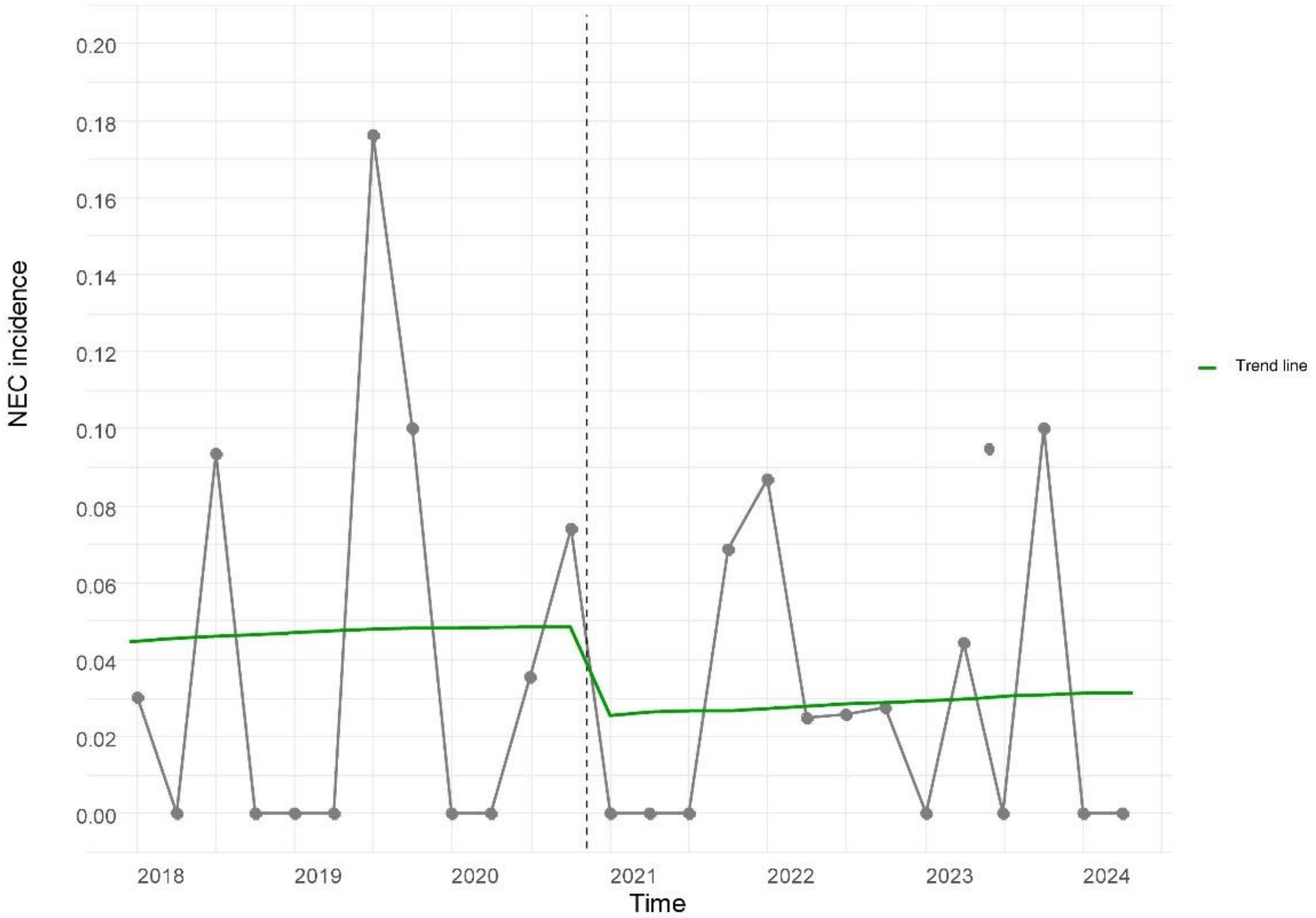

The study period spanned from January 1st, 2018, to July 1st, 2024. The grey line represents incidence for NEC over time. The green line represents the fitted trend line for the segmented interrupted time series regression model.

© 2026 Imren C et al. *JAMA Network Open*.

eTable 1. Interrupted Time-Series Results for the Study and Reference Cohorts

a. Interrupted time series results for the study cohort

Variable	β	Risk ratio (95% CI)	p-value
Pre-implementation time trend	0.009	1.01 (0.94–1.08)	0.80
Moment of implementation	-0.76	0.47 (0.21–1.00)	0.052
Post-implementation time trend	-0.014	0.99 (0.89–1.09)	0.79
Sex (male)	0.45	1.57 (1.08–2.32)	0.02
Gestational age (weeks)	-0.49	0.61 (0.54–0.69)	<0.001
Birthweight z-score	-0.084	0.92 (0.75–1.14)	0.43

b. Interrupted time series results for the reference cohort

Variable	β	Risk ratio (95% CI)	p-value
Pre-implementation time trend	0.009	1.01 (0.87–1.18)	0.88
Moment of implementation	-0.78	0.46 (0.09–2.20)	0.33
Post-implementation time trend	0.015	1.02 (0.82–1.25)	0.93
Sex (male)	-0.14	0.87 (0.41–1.88)	0.72
Gestational age (weeks)	-0.23	0.79 (0.41–1.54)	0.49
Birthweight z-score	-0.13	0.88 (0.54–1.40)	0.59

Models are Poission regression models with log link. Beta coefficients represent log risk ratios, showing the direction of the slope (positive or negative). Risk ratios are exponentiated coefficients. Time was modelled in 3-month intervals. Models were adjusted for gestational age, sex, and birthweight z-score.

© 2026 Imren C et al. *JAMA Network Open*.

eTable 2. Perinatal Baseline Characteristics for the Reference Cohort

**Characteristics**	**Pre-implementation N = 319**	**Post-implementation N = 452**
Sex, male	172 (53.9)	256 (56.6)
Gestational age, weeks + days	31.0 [30.4 – 31.6]	31.0 [30.6 – 31.4]
Birth weight, grams	1540 [1320 – 1710]	1560 [1350 – 1725]
Birth weight, z-score	0.02 [-0.57 – 0.60]	0.06 [-0.53 – 0.63]
Multiple birth	76 (23.8)	118 (26.1)
Premature rupture of membranes	52 (16.5) Missing: n = 7	106 (25.2) Missing: n = 35
Prenatal steroids, full course	174 (59.2) Missing: n = 17	212 (56.4) Missing: n = 75
Caesarian section	183 (57.9)	281 (62.4)
Apgar score, 5 minutes	8 [7 – 9] Missing: n = 6	8 [7 – 9] Missing: n = 15
Early antibiotics	233 (73.0)	333 (73.7)

Data are shown as number (%) for categorical variables and as median [25th - 75th percentile] for continuous variables for patients born before and after implementation of routine probiotic supplementation. [a]Defined as birth weight for gestational age < -2 SD (based on Fenton growth charts).

eTable 3. Unadjusted Outcomes for the Reference Cohort

**Outcome measures**	**Pre-implementation N = 319**	**Post-implementation N = 452**	***p*-value**
NEC ≥ Bell stage 2	16 (5.0)	12 (2.7)	0.12*
Medical NEC	11 (3.4)	4 (0.9)	NA
Surgical NEC	2 (0.6)	6 (1.3)	NA
Fatal NEC	3 (0.9)	2 (0.4)	NA
NEC grade 2A<br>NEC grade 2B<br>NEC grade 3A<br>NEC grade 3B	8 (2.6)<br>2 (0.7)<br>1 (0.3)<br>4 (1.3)	4 (0.9)<br>1 (0.2)<br>2 (0.4)<br>5 (1.1)	NA
Mortality, all-cause	9 (2.8)	11 (2.4)	NA
Mortality, non-NEC associated	6 (1.9)	9 (2.0)	NA
Focal intestinal perforation	-	2 (0.4)	NA
Late-onset sepsis	36 (11.3)	37 (8.2)	0.17**
Gram-negative<br>Gram-positive<br>Culture negative<br>Candida	2 (0.6)<br>14 (4.4)<br>19 (6.0)<br>1 (0.3)	7 (1.5)<br>15 (3.3)<br>13 (2.9)<br>1 (0.2)	NA

Data are shown as number (%). *p*-value for pre-post implementation comparison. *Fisher's exact test. **Pearson Chi-squared test.

© 2026 Imren C et al. *JAMA Network Open*.
