# Supplementary material for: Probiotic Implementation and Necrotizing Enterocolitis Risk in Preterm Infants
Source: JAMA Netw Open. 2026 Aug 31;9(8):e2631601. doi: 10.1001/jamanetworkopen.2026.31601 (PMC13531319; doi:10.1001/jamanetworkopen.2026.31601)
Supplement: Supplementary file 2 [file jamanetwopen-e2631601-s002.pdf]

Data Sharing Statement

Imren. Probiotic Implementation and Necrotizing Enterocolitis Risk in Preterm Infants. *JAMA Netw Open*. Published August 31, 2026. doi:10.1001/jamanetworkopen.2026.31601

Data
Data available: No

Additional Information
Explanation for why data not available: The data used in this study are pseudonymized but contain sensitive clinical information, including dates of birth, which may allow for potential re-identification. Therefore, the datasets are not publicly available. Data may be shared in aggregated form or upon reasonable request, subject to institutional and ethical approval.
